# Surface Pollen Distribution from Alpine Vegetation in Eastern Tibet, China

**DOI:** 10.1038/s41598-017-00625-7

**Published:** 2017-04-03

**Authors:** Yun Zhang, Zhaochen Kong, Zhenjing Yang, Li Wang, Xiaohong Duan

**Affiliations:** 1 State Key Laboratory of Vegetation and Environmental Change, Institute of Botany, Chinese Academy of Sciences, Beijing, 100093 China; 2 Institutes of Hydrogeology and Environmental Geology, Chinese Academy of Geological Sciences, Shijiazhuang, 050061 China; 3 University of Chinese Academy of Sciences, Beijing, 100049 China; 4 Hebei GEO University, Shijiazhuang, 050031 China

## Abstract

We explore the relationship between modern pollen spectra and vegetation patterns in the Eastern Tibet, China in order to provide information on the representation of pollen taxa and improve the general knowledge of vertical pollen transport. Forty-two modern pollen samples collected in surface soil along two altitudinal transects allowed conclusions on vertical pollen dispersal from the alpine region of Dingqing County, Changdu district in Tibet. Discriminant analyses and detrended correspondence analysis (DCA) of 24 pollen taxa were used to further discuss the difference of modern pollen spectra in these alpine vegetation zones. The surface pollen assemblage is divided into three pollen zones, such as subalpine shrub meadow, montane coniferous forest and shrub steppe with sparse trees. Altitude and precipitation are two primary factors contributing to changes in surface pollen assemblage from alpine vegetation in the eastern Tibet. Large amounts of spruce pollen at higher elevations above the timberline might be introduced from lower elevations by upslope winds. Therefore, the interpretation of spruce pollen in the fossil record must take into account long distance upward wind transport. Moreover, the destruction of coniferous forest in the study area is well illustrated in the modern pollen rain.

## Introduction

The study of modern pollen assemblage forms the basis for remodeling past vegetation and climate. Such studies became popular from the 1970s through the 1980s, and there is a large amount of literature on the subject^1–5^. Studies at this stage were concentrated on the interpretation of modern pollen assemblage at only local and small regional scales. Since the 1990s, more attention has been paid to pollen–vegetation relationships, which have become increasingly local, regional, and global concerns^6–8^. Multivariate analyses, including discriminant analysis^9^ and ordination analysis^10^, that involve the use of mathematical and statistical techniques have been used to interpret the complicated interaction between surface pollen and modern vegetation^9, 11, 12^.

The alpine zone, where the vegetation is distributed along an altitudinal gradient, is a unique ecosystem for studying the modern pollen–vegetation relationship because of its complex topography and special mechanism of pollen dispersal and transportation by air currents^7, 13^. In China, most modern pollen data have been documented in regions of low elevation, whereas few studies have been performed in higher altitudes. Of course, in certain respects, the modern pollen spectra in the alpine zone should be different from that in adjacent lowlands. Recent studies in alpine regions, such as the Tianshan Mountains, China^14^; a mountain/valley system in Niederhorn, Switzerland^13^ the Colorado Rocky Mountains, USA^15^; and the Northwestern Kunlun Mountains, China^16^, have shown that pollen spectra at high altitudes might contain pollen grains derived from vegetation growing at lower elevations; therefore, they do not necessarily reflect local vegetation, which gives us information on the mechanisms of vertical pollen transport in the alpine system.

The Qinghai–Tibetan Plateau has an area >2.3 million km^2^ and an average elevation of >4 000 m above sea level^17^. It is the largest and highest mountain plateau on the Earth and occupies most of the Tibet Autonomous Region and Qinghai. Because of its prominent features, this mountain plateau significantly influences atmospheric conditions in the Northern Hemisphere and has a great impact on pollen dispersion, preservation, transportation, and deposition. We collected and analyzed an extensive amount of modern pollen data from the Qinghai–Tibetan Plateau^12, 17–19^. The results showed that most pollen samples from this region were collected mainly from Central Tibet and seldom from Eastern Tibet. Because of mountain uplift and stream trenching, there are a lot of high mountains and valleys with great differences in altitude in the Changdu District, Tibet Autonomous Region, resulting in a distinct vertical distribution of vegetation and soil types. Moreover, the Tibetan Plateau is the least anthropologically disturbed region in China and provides a unique opportunity to study the relationships between surface pollen and modern vegetation. Therefore, it is an appropriate area where pollen rain is expected to more closely represent the “natural” vegetation, and the modern pollen–vegetation relationship derived from pollen-rain studies can provide a more realistic basis for paleovegetational reconstruction.

**Figure 1:**
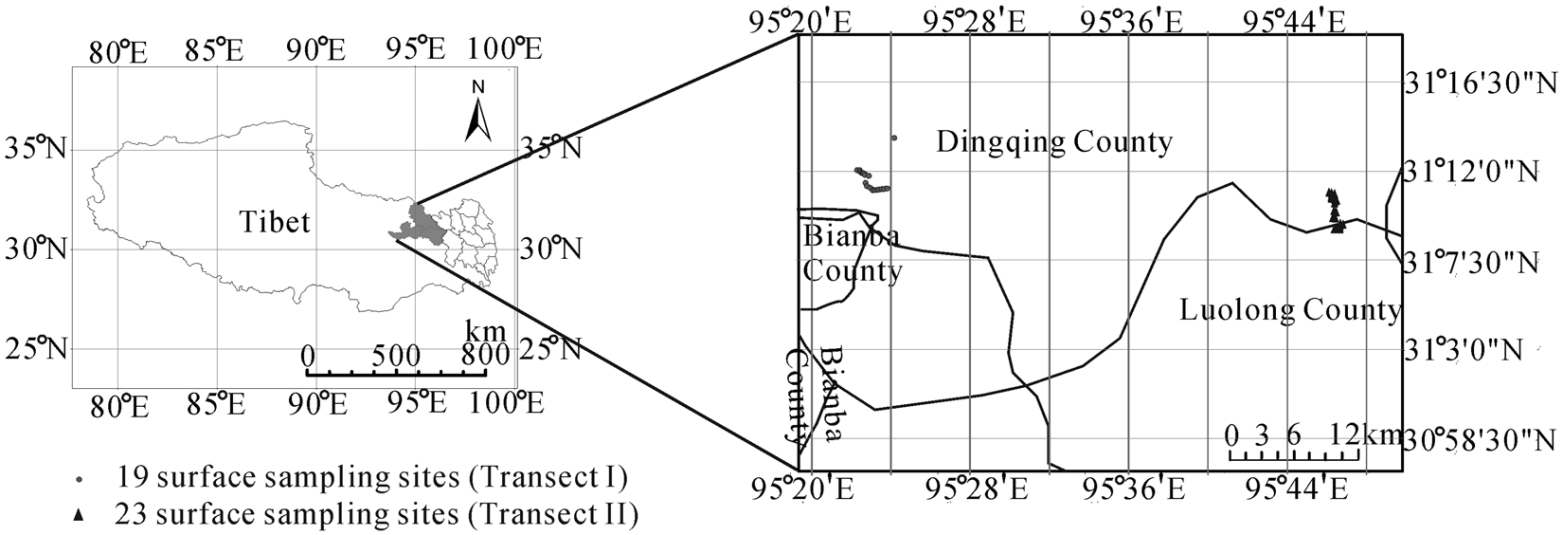
Surface pollen samples sites along an altitudinal gradient from Dingqing county in Tibet, China (Chinese map in the figure was created by ArcGIS 9.3 software, http://www.arcgis.com/features/).“Scientific Reports remains neutral with regard to jurisdictional claims in published maps”.

In this paper, we want to discuss altitudinal changes of surface pollen and vegetation in Eastern Tibet. Modern pollen samples were obtained on two altitudinal transects from the alpine area of Dingqing County, Changdu District, Eastern Tibet (Fig. 1). These data were then subjected to statistical analyses to further discuss the environmental factors that affect surface pollen assemblages in the region.

## Results

### Surface Pollen Assemblage of Different Vegetation Zones

There were 16 796 grains identified as terrestrial pollen within 49 pollen taxa. Among them, dominant arboreal pollens were *Picea* and *Betula*, but *Pinus*, Cupressaceae, *Abies* and *Salix* were occasionally found. Ericaceae was the common shrub type. *Artemisia* was the main mesic-xerophytic shrub and herb pollens. *Polygonum*, Compositae, Leguminosae and Cyperaceae dominated in mesic and hygrophyte herb pollens. Fern pollen types were Polypodiaceae and *Selaginella*.

**Figure 2:**
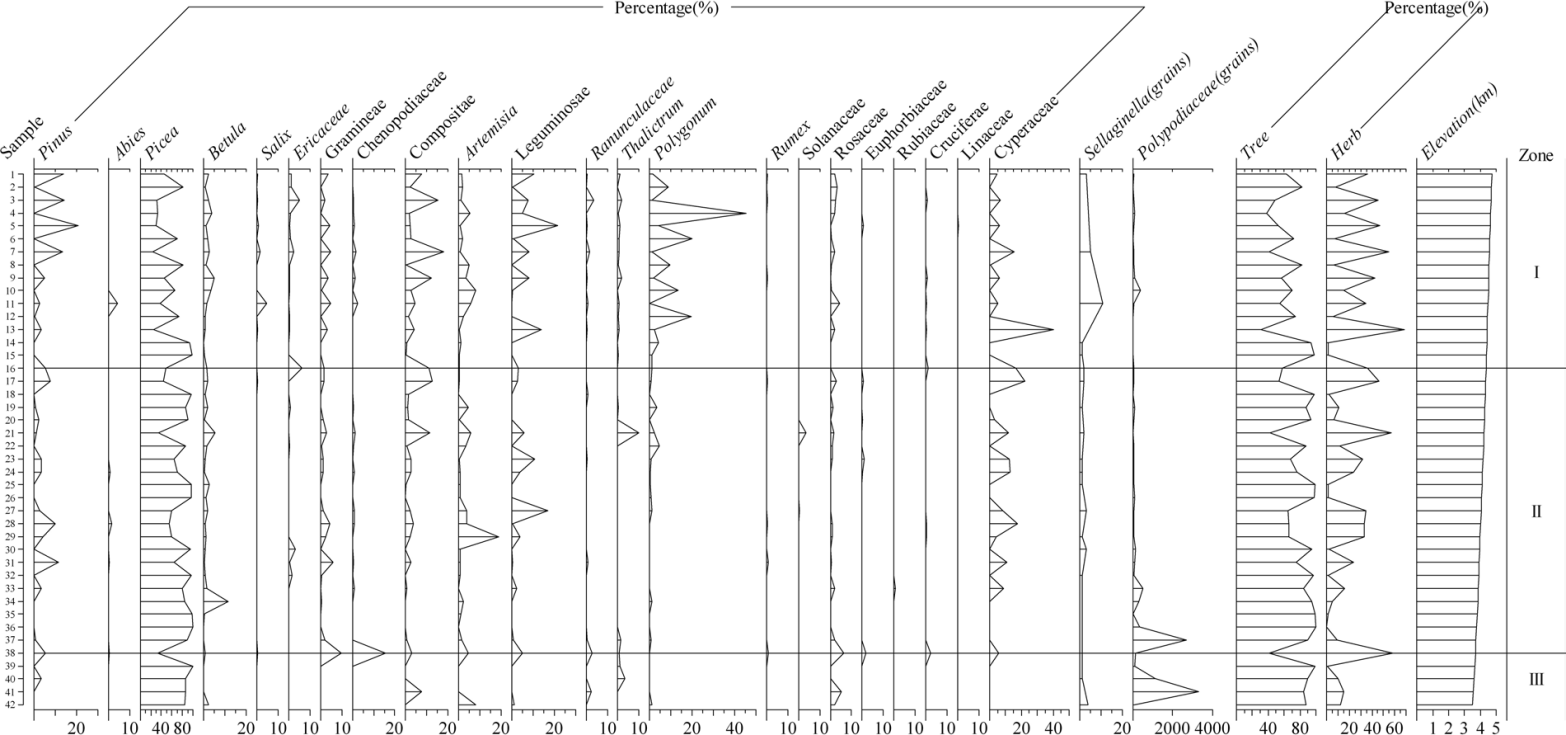
Surface pollen percentages and pollen zones of alpine area from Dingqing county in Tibet, China.

Sporopollen spectrum in this alpine area could be divided into three surface pollen zones based on modern vegetation investigation results (Fig. 2). And each zone is summarized in the following text.

Montane shrub meadow (4 740–4 320 m) (Zone I): Vegetation consist of mainly secondary shrub and grass, such as *Potentilla fruticosa*, *Potentilla glabra*, *Sibiraea angustata*, *Berberis* sp., *Caragana sinica*, *Salix* sp., and occupies the transitional areas between the forest and alpine meadows. In this zone, tree pollen, notably *Picea* (23.6–97.3%), occupied a larger proportion (average of 55.2%) in this zone. The average pollen values for shrubs and herbs were 2.26% and 26.5%. The herbaceous pollen assemblages were dominated by *Polygonum* (1.1–19.3%), Compositae (0.2–18.5%) and Legumineae (0.16–13.9%) pollen; consequently, a *Picea*–*Polygonum–*Compositae pollen combination was represented in this zone.

Montane coniferous forest (4 320–3 690 m) (Zone II): Coniferous forest with *Picea likiangensis* var. *rubescens* and *Abies squamata*, occupies the moist areas on the north-facing slope, which might be a little changed to secondary forests of *Populus davidiana* and *Betula platyphylla* after the destruction of the forest. *Juniperus tibetica* is distributed mainly on the south-facing slope in this zone. The zone was dominated by arboreal pollen (maximum value of 99.8%) with a large proportion of *Picea*. The highest *Betula* percentage (11.4%) was shown in this zone. Shrub pollen percentage was low (<2%). Higher percentages of Leguminosae (17.1%) and *Artemisia* (18.8%) were found at a middle elevation in this zone; however, their values were reduced to 2% at lower elevations. Therefore, the pollen composition in this zone can be regarded as a *Picea*–*Artemisia*–Leguminosae combination.

Shrub steppe with sparse trees (3 690–3 510 m) (Zone III): Some arboreal trees with *Picea likiangensis*, *Betula platyphylla* and *Juniperus tibetica* and shrubs consisting of *Spiraea* spp., *Lonicera rupicola* and *Potentilla glabra* occupy this zone. Percentages of arboreal pollen were still very high in this pollen zone, such as *Picea* (mean proportion of 90.1%). Mean percentage of Compositae accounted for 7.50%. *Artemisia* pollen was only 7.97%. It reflects that a *Picea*-*Artemisia-*Compositae pollen assemblage dominates this zone.

The surface pollen spectra indicate that altitude and precipitation were the primary factors affecting in surface pollen assemblage along this transect. In some zones, there are different pollen spectra in different vegetation zones. Zone II was characterized by the maximum percentage of *Picea*. Its vegetation cover was also much higher in this zone. The mean vegetation cover of *Caragana sinica* was >20% in Zone II, and their maximum pollen percentage was 21%. Surface pollen percentages provided a good indication of the vegetation types; however, surface pollen couldn’t reflect modern vegetation compositions perfectly in some zones. In Zone I, the maximum percentage of *Picea* was >90% from 4 740 to 4 320 m, which is beyond the spruce forest line.

### DCA Ordination Analysis

**Figure 3:**
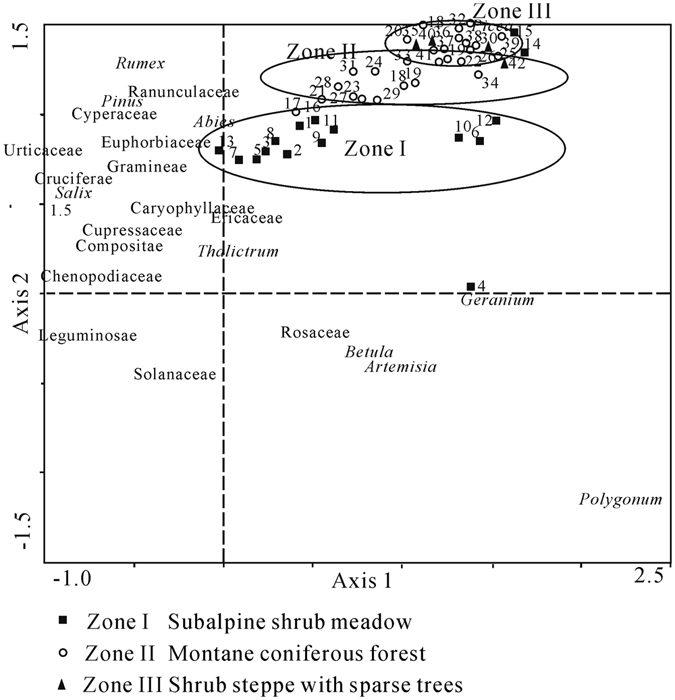
Ordination results of 42 surface pollen samples and 24 pollen taxa based on detrended correspondence analysis.

Figure 3 suggested that eigenvalues of the first two axes were 0.209 and 0.105 (78.4% of the cumulative variance), while those of the second two axes were 0.07 and 0.045, which account for only 21.6% of the cumulative variance. Thus, the first two axes are the most important environmental factors.

Ordination of the 42 surface samples suggested that the two group centroids were entirely separated (Fig. 3). Samples of subalpine shrub meadow zone were divided from those of the other zones. But there are some overlapping between montane coniferous forest and shrub steppe with sparse trees zones. Some of the overlapping samples were from the ecotonal area between these zones. It reflects the vegetation and pollen composition have the transitional character.

With respect to pollen taxa, Ericaceaea showed lower scores on the first axis, whereas *Picea* showed higher scores, indicating that the first axis represented the gradients of altitude change. Pollen types of typical desert vegetation (Chenopodiaceae) were located on one end with lower scores and those of moisture-favoring taxa (Cyperaceae and *Picea*) were located on the other end of the second axis with higher scores. The ordination result showed that the second axis represented the gradients of humidity changes; therefore, we concluded that there were two dominant factors that affect surface pollen assemblage in the region: elevation and humidity.

### Discriminant Analysis

**Figure 4:**
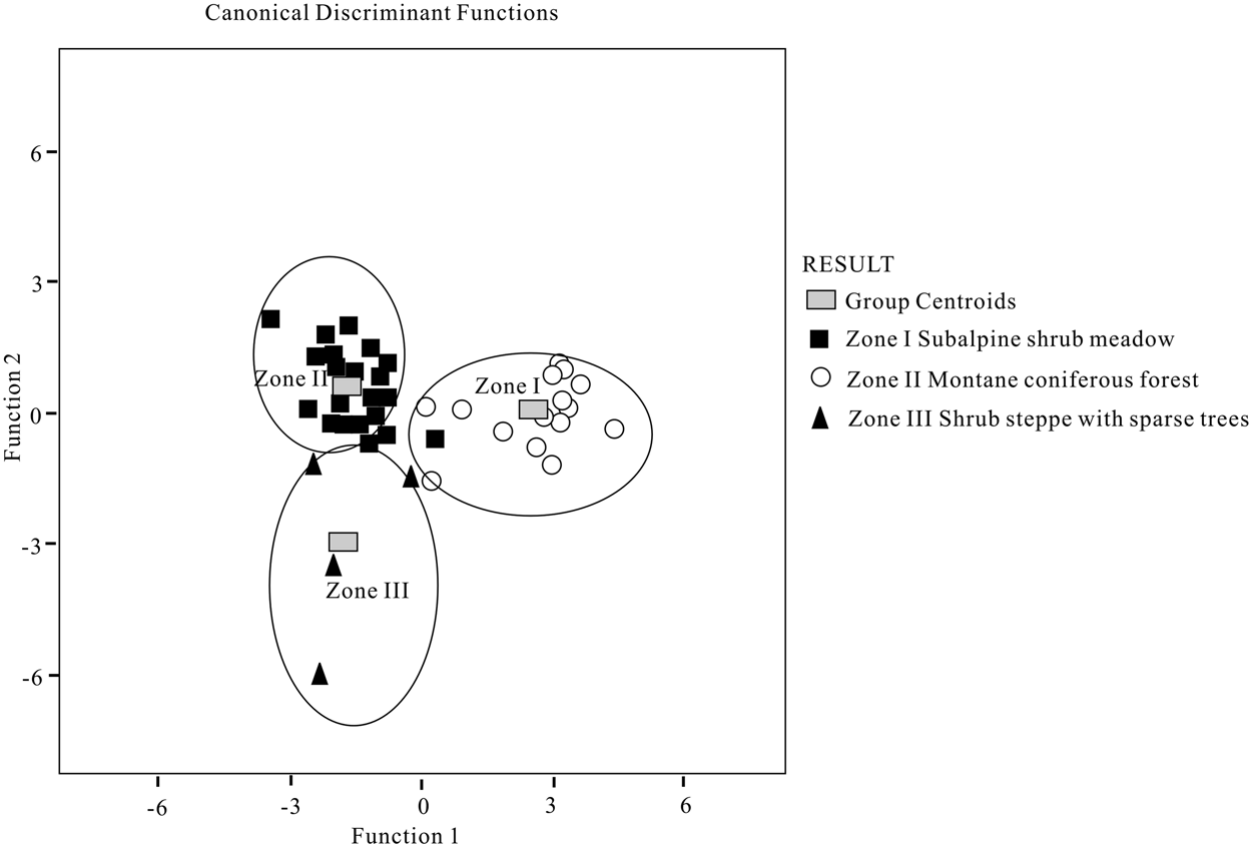
Ordination results of 42 surface pollen samples based on discriminant analysis.

Based on the regional and local vegetation characteristic in the area, we divided the 42 modern pollen data into three major priori vegetation groups, such as subalpine shrub meadow group, montane coniferous forest group and shrub steppe with sparse trees group. Results suggest that 79.7% and 20.3% of the total variance could be interpreted by two discriminant functions (Table 1). Figure 4 further shows that the three group centroids along discriminant functions 1 and 2 are separated from each other. The samples of subalpine shrub meadow separate from those of the other zones. Similarly, there are some overlapping between montane coniferous forest and shrub steppe with sparse trees zones.

**Table 1:** Coefficients in discriminant function of the 24 pollen taxa.

Pollen taxa	Function 1	Function 2
*Pinus*	0.382	−0.860
*Abies*	−1.667	0.043
*Picea*	7.833	0.713
Cupressaceae	−0.988	1.104
*Betula*	−0.862	0.260
*Salix*	4.313	−1.082
Ericaceae	0.655	0.806
Gramineae	1.347	1.863
Chenopodiaceae	−0.012	3.077
Compositae	1.739	−0.063
*Artemisia*	1.177	0.283
Leguminosae	2.632	−0.917
Ranunculaceae	−0.172	0.120
*Thalictrum*	1.405	−2.227
*Polygonum*	3.874	0.274
*Rumex*	−0.119	−0.524
Solanaceae	−0.229	1.431
Rosaceae	0.477	−0.841
Euphorbiaceae	−1.169	0.616
Urticaceae	0.512	1.235
Caryophyllaceae	1.270	1.141
Cruciferae	2.096	−3.690
*Geranium*	1.673	−0.025
Cyperaceae	3.327	0.711
Percentage variance accounted for	79.7	20.3
Cumulative percentage of variance	79.7	100.0

Table 2 suggests that samples from subalpine shrub meadow and shrub steppe with sparse trees are correctly classified. Only two out of the 22 montane coniferous forest samples are misclassified into subalpine shrub meadow vegetation and shrub steppe with sparse trees.

**Table 2:** Discriminant analysis results of the surface samples.

Actual groups	Numbers of samples	Predicted groups		
		subalpine shrub meadow	montane coniferous forest	shrub steppe with sparse trees
subalpine shrub meadow	16	14^a^(87.5^b^)	1^a^(6.25^b^)	1^a^(6.25^b^)
montane coniferous forest	22		22^a^(100^b^)	
shrub steppe with sparse trees	4			4^a^(100^b^)

Samples numbers: 42.

Misclassified samples numbers: 2.

Percentage of correctly classified samples: 95.2%.

^a^Surface samples numbers classified as that group.

^b^Surface samples percentage of classified as that group.

## Discussion

**A**s one of the dominant tree species in boreal and cold-temperate evergreen coniferous forests, *Picea*, consisting of about 16 species and 9 variables in China, is continuously distributed in the northeastern region and discontinuously distributed in the high mountains of the northern, northwestern, southwestern, and eastern regions, including Taiwan^20^. Spruce pollen has received more attention in studies of both modern pollen–vegetation relationships and paleovegetation and paleoclimate reconstructions^6, 21^; however, *Picea* pollen in surface soil is an over-representative taxa due to its bisaccate. Impacts from wind, topography, and water on the number of *Picea* pollen in surface soils has been studied on the basis of data analysis in different vegetation zones of China^21–23^. On the basis of 131 surface pollen records of spruce in Xinjiang, Northwestern China, including the Tianshan Mountains, the Altay Mountains, the Kunlun Mountains, the Tarim Basin, and the Junggar Basin, Yan *et al*.^24^ proposed that the pollen had a mean value of >30% within the spruce forest. However, the value is still >20% above the upper forest treeline, indicating an impact from the uplift of the mountain–valley wind. The strong wind-driven uphill transport of arboreal pollen to higher elevations because of atmospheric processes was first discovered in the European Alps^25, 26^ and has also been recorded in the mountains of Ecuador^27^, Switzerland^13^, Canada^28^, and Argentina^29, 30^. In China, the phenomenon was also observed in Yunnan, Southwestern Sichuan Province^31^ and even on the Tibetan Plateau^32^.

*Picea* was dominant in modern pollen data in the study area. The mean pollen percentage of spruce was >55% beyond the upper spruce forest limit. It was thought to reflect upslope wind-transported pollen transportation, which has been discussed in other studies in Tibetan^32, 33^. Because of westerly jet of the plateau and the topography of alpine gorges in the Changdu area, mountain–valley winds are very strong in this area. Mountain–valley wind is characterized as a down-valley wind at night and ascending flows during the day. The Tibetan Plateau has a well-developed, thermally driven, mountain–valley wind system^34^. From 2000 to 2004 and from 29 May to 29 June, 2006, a strong down-valley wind was observed along the northern slope of Mt. Everest^35, 36^; however, there was a strong daytime up-valley wind in the Kali Gandaki Valley of the Himalayas^37^.

**Figure 5:**
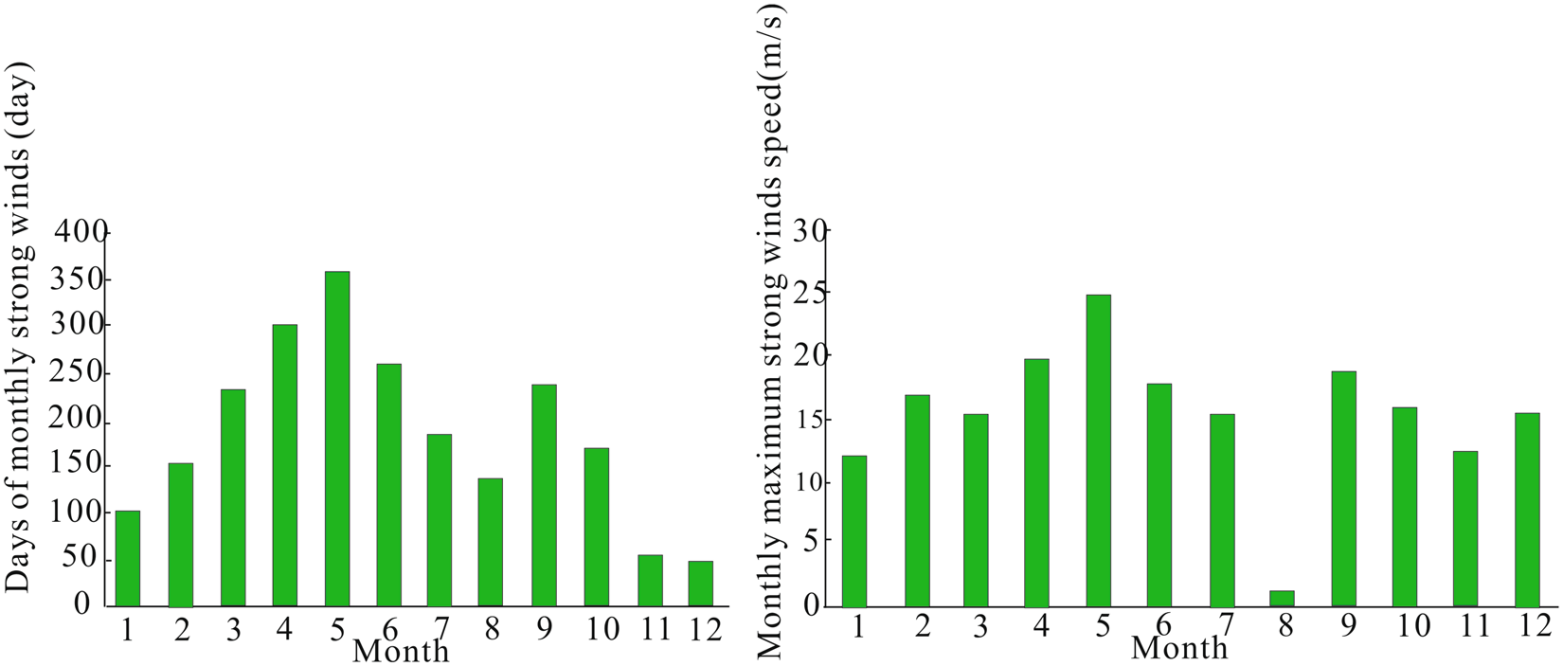
Total days of monthly gales and monthly maximum gales speed from Dingqing weather station (1970–2009).

The meteorological data (1970–2009) of wind speeds and wind directions were obtained from the Dingqing weather station (31°25′N, 95°36′E). The monthly average wind velocity at the station varied from 1.5 m/s to 2.1 m/s, but strong winds were frequently observed in the area. Figure 5 shows the maximum total number of days, with strong winds from 1970 to 2009, as recorded from April through June, which accounted for 45% of number of days in the entire year. Second, extreme wind speeds >18 m/s were also observed from April to June. Moreover, the dominant wind direction of extreme winds was northwest. Hence, in this area, northwest daytime upslope wind dominates in spring and summer. In addition, modern pollen data from the Tianchi Weather Station (1 980 m), the Fukang Research Station of the Chinese Academy of Sciences (460 m), and the Beishawo Field Station (400 m) in Xinjiang of China from July 2001 to July 2002 demonstrated that the pollination season for spruce is early June^14^. Therefore, during the day, the prevailing wind direction is northwest and wind speed is stronger than that at night, resulting in more spruce pollen grains at higher elevations being carried from the lowlands. However, night-time wind speeds are somewhat slower; therefore, no actual down-slope pollen dispersal is found. Accordingly, the upward transportation of spruce pollen from lower elevations interferes with pollen assemblage at the higher elevations.

Because *Betula* is wind pollinated and produces abundant, well-dispersed pollen, it is often over-represented in the pollen record^38, 39^. However, many previous studies have also shown that *Betula* pollen is equi-represented in the pollen rain^3, 40^. High percentages of *Betula* (up to 40%) undoubtedly indicate the local presence of the genus^41^. The consistent presence of *Betula* at >5% in modern pollen spectra might also reflect the presence of birch woodland^42–44^.

Therefore, *Betula* pollen showed a strong linear relationship with *Betula* tree abundance, and the highest *Betula* values of 11.4% from Zone II in the study area corresponded closely to the distribution of the secondary forest of *B*. *platyphylla* after the destruction of the coniferous forest; this accurately illustrated human activities in the modern pollen rain. The mean pollen percentage of *Betula* was >2% m in vegetation Zones I and II, reflecting upslope wind transport.

In Zone I, the presence of *Abies* pollen even at low percentages (4.54%) is most likely linked to the existence of *A*. *squamata* above the spruce forest zone. Lu *et al*.^45^ also stated that fir forests generally grow above spruce forests. Although *J*. *tibetica* was the common tree in the south-facing slope of the study area, its pollen abundance was very low. Cupressaceae pollen may be somewhat over-represented, with low pollen productivity and poor pollen dispersal; therefore, Shen^46^ proposed that it is excluded from the major pollen types because it is poorly preserved in fossil pollen spectra.

## Conclusion

An analysis of modern pollen rain along two altitudinal transects from alpine vegetation in Eastern Tibet, China, indicated that changes in altitude and precipitation were the primary factors that accounted for distinct surface pollen spectra. *Picea* pollen was observed in the surface soils of all vertical vegetation belts in the region, implying effective upward transport of spruce pollen from the coniferous forest zone by the upslope wind. The complicated local atmospheric conditions had a large impact on surface pollen dispersion and transportation in the regions. Thereby, the pollen spectra in lower elevations representing a local aspect whereas those in higher elevations recorded the vegetation types from the low altitude. Researchers must therefore pay more attention to vegetation reconstruction based on pollen samples with high spruce pollen concentration in the stratum of the alpine vegetation zone in Eastern Tibet, China. Moreover, the modern pollen data also documented the destruction of the coniferous forest in the study area; this indicated that human activity can change the natural features of pollen assemblage.

## Materials and Methods

### Study Area

Dingqing County (Fig. 1) (31°1′N to 32°21′N, 94°39′E to 96°17′E) in Eastern Tibet is located in the western section of Changdu District and along the southern slope of the Taniantaweng Mountains. It has a semi-humid monsoon climate with a mean annual temperature of approximately 8–12 °C, mean July temperature of approximately 16 °C, annual accumulated temperature (≥10 °C) of 1 500–4 200 °C, annual theoretic evaporation of 1 538 mm, and annual precipitation of approximately 600–800 mm^47, 48^. From top to bottom, it displays a distinct elevational distribution of vegetation^47, 49^ (sparse alpine cushion, alpine meadow, alpine shrub to montane shrub meadow, montane coniferous forest, and shrub steppe with sparse trees).

### Vegetation Investigation and Samples Collection

We collected modern pollen soil samples (Fig. 1) along two transects in Dingqing County, Changdu District, Tibet. For the first transect (Transect I), 19 samples were connected from 4740 m to 3 693 m. The second transect (Transect II) was located at intervals of 20–100 m from 4700 m to 3510 m.

We treated surface pollen samples in the laboratory with heavy liquid and acetolysis after chemical treatments^50^. Using 400x magnification with an Olympus microscope, we examined two to three slides of each sample and counted roughly 300 terrestrial pollen grains. Unknown and indeterminate pollen grains, such as broken, concealed, or corroded pollen, were excluded. Percentages of pollen were calculated based on the total pollen sum (excluding spores) (Fig. 2), using the Tilia/TiliaGraph software^51^.

### Statistical Analysis

We selected *Pinus*, *Abies*, *Picea*, Cupressaceae, *Betula*, *Salix*, Ericaceae, Gramineae, Chenopodiaceae, Compositae, *Artemisia*, Leguminosae, *Polygonum*, Ranunculaceae, Rumex, Solanaceae, Rosaceae, Euphorbiaceae, Urticaceae, Caryophyllaceae, Cruciferae, Geranium, Thalictrum, and Cyperaceae for discriminant analysis and detrended correspondence analysis (DCA). Statistical analysis was processed using the CANOCO software package^52^.
